# *SCENTinel 1.0*: Development of a Rapid Test to Screen for Smell Loss

**DOI:** 10.1093/chemse/bjab012

**Published:** 2021-03-27

**Authors:** Valentina Parma, Mackenzie E Hannum, Maureen O’Leary, Robert Pellegrino, Nancy E Rawson, Danielle R Reed, Pamela H Dalton

**Affiliations:** 1 Department of Psychology, Temple University, 1701 N 13th Street, Philadelphia, PA, USA; 2 Monell Chemical Senses Center, 3500 Market Street, 19104, Philadelphia, PA, USA

**Keywords:** anosmia, hyposmia, prediction, smell loss, smell test

## Abstract

Commercially available smell tests are primarily used in research or in-depth clinical evaluations and are too costly and time-consuming for population surveillance in health emergencies like COVID-19. To address this need, we developed the *SCENTinel 1.0* test, which rapidly evaluates 3 olfactory functions: detection, intensity, and identification. We tested whether self-administering the *SCENTinel 1.0* test discriminates between individuals with self-reported smell loss and those with average smell ability (normosmic individuals) and provides performance comparable to the validated and standardized NIH Toolbox Odor Identification Test in normosmic individuals. Using Bayesian linear models and prognostic classification algorithms, we compared the *SCENTinel 1.0* performance of a group of self-reported anosmic individuals (*N* = 111, 47 ± 13 years old, *F* = 71%) and normosmic individuals (*N* = 154, 47 ± 14 years old, *F* = 74%) as well as individuals reporting other smell disorders (such as hyposmia or parosmia; *N* = 42, 55 ± 10 years old, *F* = 67%). Ninety-four percent of normosmic individuals met our *SCENTinel 1.0* accuracy criteria compared with only 10% of anosmic individuals and 64% of individuals with other smell disorders. Overall performance on *SCENTinel 1.0* predicted belonging to the normosmic group better than identification or detection alone (vs. anosmic: AUC = 0.95, specificity = 0.94). Odor intensity provided the best single-feature predictor to classify normosmic individuals. Among normosmic individuals, 92% met the accuracy criteria at both *SCENTinel 1.0* and the NIH Toolbox Odor Identification Test. *SCENTinel 1.0* is a practical test able to discriminate individuals with smell loss and will likely be useful in many clinical situations, including COVID-19 symptom screening.

## Introduction

The COVID-19 pandemic has shown us how vulnerable we are to diseases that find an entry point in the olfactory system (Brann et al. 2020; Cooper et al. 2020; Pellegrino et al. 2020;Rodriguez et al. 2020). Despite the sudden onset of smell loss that is common in people with COVID-19 (Menni et al. 2020; Roland et al. 2020; Yan et al. 2020), the sense of smell is rarely evaluated in routine medical care, an omission that can have significant negative clinical implications (such as missed early identification of neurodegenerative disorders, lack of development of treatment options; Neuland et al. 2011; Croy et al. 2014; Boesveldt et al. 2017; Erskine and Philpott 2020). Failure to see the mainstream clinical potential of evaluating the sense of smell is due to both theoretical and practical factors. Smell may be viewed as unimportant or as a vestigial sense despite a wealth of evidence to the contrary (McGann 2017). As a result, olfactory function is rarely assessed until an individual experiences a significant—often complete—smell loss. In addition, there is widespread lack of both primary and specialty physicians able to evaluate “normal” olfaction apart from questionnaires, leaving the diagnosis of olfactory loss to a few specific specialties. Such shortage of widespread olfactory assessments across the life span likely results in underestimating the true prevalence of smell loss in the general population. However, as COVID-19 has revealed, such routine and rapid smell tests for population surveillance are needed. A recent meta-analysis highlights the sensitivity of direct measures of smell compared with self-reports (Hannum et al. 2020). Unless it is measured directly, many people do not realize their sense of smell is partially reduced, which might help to explain why three-quarters or more of people with COVID-19 self-report no symptoms at all (Letizia et al. 2020; Petersen and Phillips 2020). However, infection with COVID-19 is not the only cause of olfactory disorders. Indeed, anosmia (total loss of smell) and hyposmia (decreased ability to smell) can be caused by many respiratory viruses, including the common cold (Temmel et al. 2002; Pellegrino et al. 2017; Cavazzana et al. 2018), as well as sinonasal disease, neurodegenerative disorders, and head trauma among others (Damm et al. 2002; Dalton 2004; Nordin and Brämerson 2008; Doty 2017; Hummel et al. 2017).

Current smell tests do not meet requirements for population surveillance. A number of validated smell tests are commercially available (Doty et al. 1984, 1996; Hummel et al. 1997; Kondo et al. 1998; Duff et al. 2002; Choudhury et al. 2003; Jackman and Doty 2005; Dalton et al. 2013; Croy et al. 2015; Rawal et al. 2015; Liu et al. 2020). These tests are suitable for research and in-depth clinical testing, yet they do not meet the scientific and practical needs for population surveillance (e.g., speed and low cost). There are several ways to measure olfaction, to see if a person can detect/discriminate the presence of an odorant or can correctly identify the odorant. Using a scale to rate the intensity of an odorant offers an additional measure of sensitivity, which while often used in research, is not regularly assessed in commercial smell tests. Most existing smell tests include only a single olfactory task: odor identification (Doty et al. 1984; Duff et al. 2002; Jackman and Doty 2005; Dalton et al. 2013; Rawal et al. 2015). Although the most popular, odor identification is also the olfactory skill most sensitive to cognitive deficits (e.g., verbal memory impairment; Wilson et al. 2006; Hedner et al. 2010), which can result in impaired performance for non-sensory reasons. Odor identification alone may fail to detect reduction in intensity (especially among young people, who contrary to a more elderly population, may have lost much ability to smell but nevertheless retain enough ability to guess the odorant’s quality). Additionally, odor intensity, even when self-reported, has proven to be the most predictive symptom indicator of a COVID-19 diagnosis (Gerkin et al. 2020). Indeed, either an odor detection, discrimination, or identification test can reveal whether an individual suffers from functional anosmia. Yet, if their sense of smell is only partially diminished (hyposmia) or distorted (parosmia), testing different smell functions will reveal divergent results. For example, a person with hyposmia may detect and discriminate a target odor depending on concentration and, if so, may identify an odor’s quality. However, a person with parosmia may detect and discriminate an odor but fail to identify it. Indeed, measuring different olfactory functions reveals response patterns commonly associated with different etiologies (Whitcroft et al. 2017). Therefore, there is a need to develop a smell test that rapidly assesses multiple olfactory functions in order to provide an assessment of smell loss that can be optimized for routine use and population surveillance.

Large-scale deployment of a smell test would be ideal for population surveillance. At least 6 considerations are important for large-scale deployment of a smell test: (a) fast execution and administration without trained personnel, (b) use of easily identifiable odorants, (c) several test versions to allow for people to take the test frequently, (d) uniform delivery of odorants across sessions, (e) protection from physical contamination while taking the test, and (f) correct answers that are not easy to guess. Speed is important because smell testing, especially for population surveillance, such as for building admittance, must be fast. Odorant choice is important because the odorants must be familiar within the cultural or geographic context where the test is used, to minimize misattributions that do not depend on the ability to smell (Rabin and Cain 1984; Ayabe-Kanamura et al. 1998). Odorants should not have a pungent component due to trigeminal activation (such as mint and cinnamon) because they can be detected by anosmic individuals (Laska et al. 1997). The number of odorants is important because the test could be repeatedly taken (for instance, each day for several weeks), and it should include enough odorants that people do not give rote answers. Uniformity in how the odorant is delivered is important (e.g., use of odorant pens), and they should be easily accessed without tools (such as coins often used for scratch-and-sniff tasks) and without introducing new sources of variation (such as unequal scratching when releasing the odorant). Avoiding physical contamination is important, and participants cannot share the same olfactory stimulus (e.g., single-use, disposable tests to reduce the transfer of potential pathogens from nose to hand). Finally, the test must be robust against guessing.

To meet these 6 criteria, we designed *SCENTinel 1.0* (a portmanteau of “scent” and “sentinel”). The self-administered test rapidly assesses 3 components of olfactory function: odor detection, intensity, and identification. To assess the performance of *SCENTinel 1.0*, we have conducted a quantitative cross-sectional study. The objective of the present research was to (i) evaluate the ability of *SCENTinel 1.0* to discriminate between individuals who self-reported as suffering from anosmia or as normosmic individuals and to (ii) determine the performance of *SCENTinel 1.0* compared with a validated and standardized smell test (NIH Toolbox Odor Identification Test) in a normosmic group.

We hypothesized that:

(i) Normosmic individuals will meet the *SCENTinel 1.0* accuracy criteria at a higher rate than the anosmic group and individuals with other olfactory disorders;

(ii) In the normosmic group, overall *SCENTinel 1.0* performance is comparable to the performance on the NIH Toolbox Odor Identification Test.

## Materials and methods

The materials, procedures, hypotheses, and preanalysis plan were all preregistered and are available in the Open Science Framework Repository (Parma et al. 2020). Additional analyses (prognostic classification analyses) are marked as exploratory in this article.

### Components of *SCENTinel 1.0*

*SCENTinel 1.0* is rapid and is less expensive than the current commercially available validated smell tests. It measures odor detection, intensity, and identification based on evaluation of a single odorant. Here, we assessed *SCENTinel 1.0*, a version that used a flower odor (Givaudan; perfume compound with 2-phenylethanol [CAS No. 60-12-8] as the main component). *SCENTinel 1.0* comprises 3 patches, created with the Lift’nSmell technology (Scentisphere), glued via an adhesive, only one of which contains an odorant (Supplementary Figure S1). This technology prevents cross-contamination of odor to the “blank” patches on the same card (imperative for an accurate odor detection test), promotes standardization of odor delivery across cards and odors (imperative for an accurate odor intensity test), and limits residual odor in the air after the test (imperative for accurate odor identification).

To complete the fulfillment of the scientific and practical criteria above, *SCENTinel 1.0* includes 2 olfactory functions that can be objectively assessed to yield a falsification metric and enable the ability to calculate the probability of meeting the test’s accuracy criteria in the absence of smell ability. The *odor detection subtest* has a guessing probability of 33%. The *odor intensity subtest* relies on the subjective experience of the participant and cannot be directly falsified. Intensity was included because a cutoff rating (<20 on a 1–100 scale; Gerkin et al. 2020) signaled a likelihood of COVID-19-associated smell loss, particularly for an odorant generally perceived as moderate to strong, and we determined this metric to be useful for tracking an individual’s smell function over time (i.e., identifying changes with repeated testing). The *odor identification subtest* comprises 2 possibilities: the first attempt, which is a four-alternative forced-choice task with guessing probability of 25%, and a second attempt for those who failed the first attempt, which is a three-alternative forced-choice task with guessing probability of 33%. To allow for comparability, we used the NIH Toolbox Odor Identification Test flower distractors (Dalton et al. 2013). For full instructions for *SCENTinel 1.0*, see the Procedures section; Table 1 shows the possible response patterns and accuracy matrix for *SCENTinel 1.0*.

**Table 1.** *SCENTinel 1.0* accuracy matrix: potential response patterns and guessing probabilities

			Identification		
Response pattern #F	Detection	Intensity (range 1–100)	First attempt	Second attempt	*P*(ch)
1	Correct	>21	Correct	NA	0.07
2	Correct	≤20	Correct	NA	0.02
3	Correct	>21	Incorrect	Correct	0.07
4	Correct	≤20	Incorrect	Correct	0.02
5	Correct	>21	Incorrect	Incorrect	0.13
6	Correct	≤20	Incorrect	Incorrect	0.03
7	Incorrect	>21	Correct	NA	0.13
8	Incorrect	≤20	Correct	NA	0.03
9	Incorrect	>21	Incorrect	Correct	0.13
10	Incorrect	≤20	Incorrect	Correct	0.03
11	Incorrect	>21	Incorrect	Incorrect	0.26
12	Incorrect	≤20	Incorrect	Incorrect	0.07

Note: Gray-shaded row: accurate response patterns; #, response pattern number. Detection is by a triangle test. “First attempt” is a four-alternative forced choice. “Second attempt” is a three-alternative forced choice. *P*(ch), probability of an outcome by chance.

### Participants

**Figure 1.**
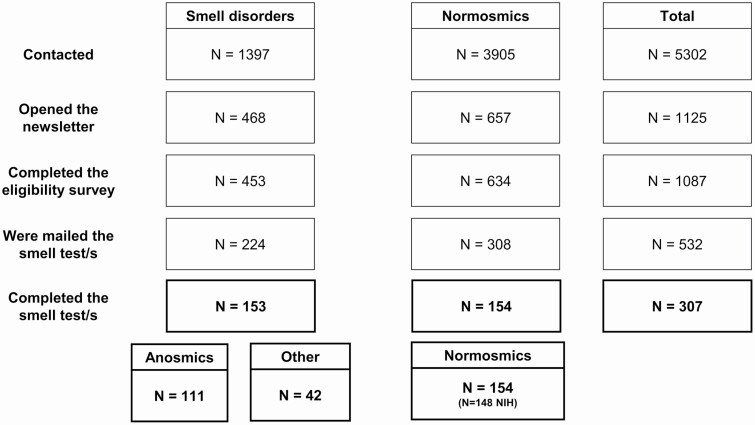
Sample description by group. Other: participants who self-reported other smell disorders. NIH: Normosmic individuals who completed the NIH Toolbox Odor Identification Test.

Eligible participants were recruited via an electronic flyer distributed through the Monell Newsletter, allowing the enrollment of normosmic subscribers and subscribers with different forms of self-reported smell loss (Figure 1). Volunteers completed an eligibility survey (Appendix) in which they reported their age (inclusion criteria: 18–75 years old, 257 excluded), whether they had access to a smart device (phone or tablet) or a computer (6 excluded), and whether they were currently residing in the United States (121 excluded). While these individuals may have been more aware of smell issues than the general population, to the best of our knowledge, none had participated in any studies utilizing the NIH Toolbox Odor Identification Test.

A total of 532 *SCENTinel 1.0* tests were distributed by mail on a first-come, first-served basis; 308 participants reporting no history of smell problems received 1 *SCENTinel 1.0* test and 1 NIH Toolbox Odor Identification Test (Dalton et al. 2013). Participants with self-reported, preexisting forms of smell loss (*N* = 224) received 1 *SCENTinel 1.0* test only; they were not asked to complete the NIH Toolbox Odor Identification Test to limit the emotional burden generated by participating in smell tasks. Participants were also invited to take *SCENTinel 1.0* (and the NIH Toolbox Odor Identification Test, if provided) on the same day they were scheduled to have a COVID-19 PCR test. We then asked them to report the results of the COVID-19 PCR test via survey when the outcome was known. Only 3 participants took the smell test/s and the COVID-19 PCR test on the same day; given the low numerosity, we excluded this variable from analyses. The completion rate of those who were sent a smell test was 58%, with a final sample size of participants who consented and participated in the study comprising 154 normosmic adults, 111 anosmic individuals, and 42 participants with other smell disorders (fluctuations [*N* = 5], hyposmia [*N* = 23], parosmia [*N* = 5], other [*N* = 4], COVID-related smell loss [*N* = 3]). Statistical power was insufficient to contrast the performance of the different “other smell disorders” subgroups, therefore no separate statistical analyses were performed on this factor. Table 2 describes the demographics of the sample. Among normosmic participants, 148 also completed the NIH Toolbox Odor Identification Test.

**Table 2.** Description of the final sample that completed *SCENTinel 1.0*

		Anosmic	Other smell disorders	Normosmic
Age (yo)	Mean ± SD	47 ± 13	55 ± 10	47 ± 14
	Range	19–72	32–69	20–74
Sex	F (%)	79 (71%)	28 (67%)	114 (74%)
	M (%)	32 (29%)	14 (33%)	40 (26%)
	Prefer not to say (%)	0	0	0
Race/ethnicity	Asian (%)	3 (3%)	0	12 (8%)
	Black (%)	3 (3%)	1 (2%)	5 (3%)
	Hispanic	2 (2%)	0	5 (3%)
	Native Hawaiian (%)	0	0	1 (1%)
	White (%)	100 (90%)	38 (90%)	128 (83%)
	Other (%)	2 (2%)	2 (5%)	1 (1%)
	Prefer not to say (%)	1 (1%)	1 (2%)	1 (1%)
*N* Total		111	42	154

Note: yo, years old; SD, standard deviation.

### Procedures

The study started on 4 September 2020 and was completed by 15 September 2020. The study was approved by the University of Pennsylvania Institutional Review Board (protocol no. 844425) and complied with the Declaration of Helsinki. During this time, participants were contacted via the Monell newsletter mail list and completed a 10-question online eligibility survey (Appendix). Subscribers to the Monell newsletter mail list include volunteer leadership; academic, industry, and organizational partners; donors; individuals with health-related interest in the research conducted at Monell; and individuals who have attended Monell events. Participants provided consent using an approved online consent form, via their smart device or computer. If they were not eligible or if they responded after the target number of participants had been enrolled, they were thanked and informed that they would not be enrolled in the study (*N* = 555; Figure 1). If, on the contrary, they were deemed to be eligible and tests were still available, they received 1 or 2 smell tests via mail, depending on their anosmic/normosmic self-report status. Once participants received the test, they were instructed to complete them within the next 14 days. Participants used a QR code or a web address to access the REDCap survey (Harris et al. 2019) used to record self-reports on demographic data (age, gender, ethnicity) and preexisting smell and taste loss, and to receive instructions to complete *SCENTinel 1.0* and the NIH Toolbox Odor Identification Test, if provided. To complete *SCENTinel 1.0*, the instructions were to consecutively open 1 odor patch at a time, smell each patch, and reseal; (a) choose the patch with the strongest odor; (b) rate the intensity of the odor on a visual analog scale from 0 (no smell) to 100 (very strong smell); and (c) select the best verbal and visual label for the odor among 4 options provided. Participants who gave an incorrect response to (c) were instructed to try again to identify the odor, this time among the 3 remaining options. No additional feedback was provided on the accuracy of the odor identification after the second attempt. Participants who also completed the NIH Toolbox Odor Identification Test (self-reported normosmic individuals) were instructed to scratch and sniff each of the 9 odors included in the NIH Toolbox Odor Identification Test and identify among 4 visual and verbal options which one corresponded to the odor smelled. Subsequently, the participants completing the NIH Toolbox Odor Identification Test could opt-in to answer questions regarding their health status, with particular reference to COVID-19 and other respiratory illnesses. The answers to those questions are irrelevant to the main hypotheses of this study and will be reported in a separate, future manuscript. Although no formal data were collected on the completion time of *SCENTinel 1.0* in the present sample, pilot participants (*N* = 10, 9 F, 27–65 years old) reported that the test takes ~2 min to complete when including the demographic questions and <1 min to complete the *SCENTinel 1.0* subtests.

### Statistical analyses

This cross-sectional design includes the between-subject factor “smell ability” (anosmic, other smell disorders, and normosmic individuals) and the following within-subject factors: meeting the accuracy criteria within each subtest of *SCENTinel 1.0* (odor detection, intensity, identification), as well as the *SCENTinel 1.0* overall accuracy criteria (Table 1), and the scores on the single items and the total score for the NIH Toolbox Odor Identification Test.

Each *SCENTinel 1.0* subtest returns one of the following responses: odor detection accuracy (correct/incorrect); odor intensity (above/below a cutoff of 20); and odor identification among 4 given options (correct/incorrect) or, if the first response is incorrect, among the 3 remaining options (correct/incorrect). The NIH Toolbox Odor Identification Test returns 2 scores: the official scoring (anosmia ≤3; hyposmia = 4–6; normosmia ≥7; Dalton et al. 2013) and a binarized version of the official score to enable direct comparison with the *SCENTinel 1.0* accuracy criteria (anosmia ≤4; normosmia ≥5). The binarized score has been used in the present analyses.

We used a sequential Bayes factor design (SBFD) with maximal *N*, as suggested by Schönbrodt et al. (2017). This maximizes the probability of obtaining the desired level of evidence and a low probability of obtaining misleading evidence. Additionally, this SBFD design requires on average half the sample size compared with the optimal null hypothesis testing fixed-*n* design, with comparable error rates (Schönbrodt et al. 2017). The desired grade of relative evidence for the alternative versus the null (BF_10_) hypothesis is set at BF_10_ > 6 (moderate evidence) for H_1_ and BF_01_ > 3 for H_0_ (anecdotal evidence). Based on a conservative Cohen’s *d* = 0.5, we have specified a minimum sample size per group of *n*_0_ = 43. Once *n*_0_ is reached, the BF will be computed on the existing data. BF computation will continue after every participant is added (in the smallest or slowest accumulating group at that time) until the threshold of H_1_ or H_0_ is reached, at which point sampling will cease. The main driver of the stopping rule is, however, a time limit (15 September). To test our hypotheses and explore covariate effects (age, sex, ethnicity), we employed Bayesian linear mixed models using the *BayesFactor* package (Morey et al. 2018) in the R Environment for Statistical Computing (R Core Team 2020). For analyses, given the unequal distribution of the data across categories in the ethnicity variable, we have binarized the responses as White/non-White. To assess the differences in accuracy among tests and subtests, we have employed Bayesian and parametric tests for equality of proportions with or without continuity correction.

In addition to the preregistered analyses, we have applied machine learning prognostic classification algorithms to confirm the ability of *SCENTinel 1.0* to discriminate anosmic and normosmic individuals, as well as individuals with other smell disorders. We removed the second trial of *SCENTinel 1.0*’s odor identification from the classification, given the high number of missing values and the challenges of imputation in those conditions. No imputation procedure was then required for the rest of the database. A one-hot encoding was applied to all categorical variables (sex and ethnicity) to produce binary indicators of category membership. Model quality was measured using receiver operating characteristic (ROC) area under the curve (AUC). We also report specificity, sensitivity, positive predictive value, and negative predictive value based on the model that optimizes classification on unseen data among random forest, linear, and radial small vector machine, regularized linear regression (Elastic net), and linear discriminant analysis (LDA). Cross-validation (number = 10, repeat = 5) was performed on the training set (80% of the sample), and validation was completed on the remaining, withheld data (20%). The model that provided the best classification AUC between anosmic and normosmic on the withheld data was LDA, which we report and discuss in the main text. The data and analysis script are available in the Supplementary material and will be publicly available on OSF (https://osf.io/5d7kx/) upon publication.

## Results

### *SCENTinel 1.0* discriminates anosmic from normosmic individuals

**Figure 2.**
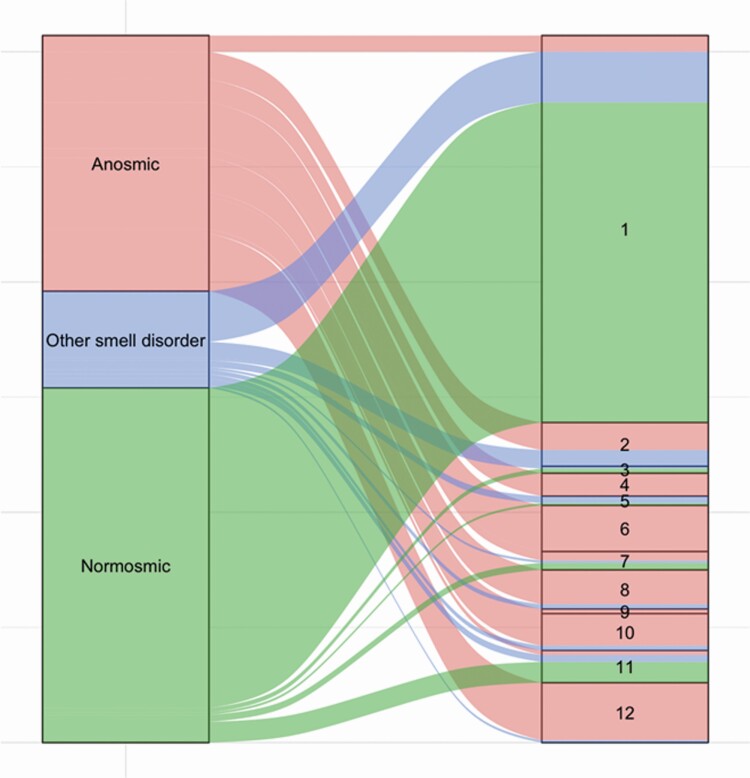
*SCENTinel 1.0* response patterns by smell group (anosmic individuals = red; other smell disorders = blue; normosmic individuals = green). Response patterns 1, 3, 5, and 7 met the SCENTinel accuracy criteria.

As expected, only a small group of anosmic individuals (*N* = 11, 10%) met the accuracy criteria for *SCENTinel 1.0*. In contrast, the majority of individuals with other smell disorders (*N* = 27, 64%) and the vast majority of normosmic individuals met the accuracy criteria for *SCENTinel 1.0* (*N* = 145, 94%). As reported in Figure 2 and Supplementary Table S1, participants from the 3 groups primarily had different response patterns in completing *SCENTinel 1.0*. In the anosmic group, 23% of participants failed to meet the accuracy criteria for any of the subtests, 41% for 2 of the 3 subtests, and only 11% failed to meet the accuracy criterion for odor intensity (i.e., reported intensity above 20/100). In the other smell disorders group, 17% of participants failed to meet the accuracy criterion for odor intensity, 17% for 2 subtests, and only 2% for all 3 subtests.

The combined accuracy at all 3 subtests significantly discriminated the performance across the 3 groups. In particular, in this sample, the odor intensity subtest demonstrates a perfect ability to identify normosmia (Table 3), as 100% of participants reported an intensity rating over the cutoff of 20. The only subtest that does not significantly discriminate between the performance of the 3 groups is the second attempt at odor identification, which was used by only 32 participants across the 3 groups (Table 3). No effects of age, sex, or ethnicity across groups were revealed for any of the *SCENTinel 1.0* subtests (Supplementary Table S2). A marginally moderate effect of age can be found in the performance of the first identification subtest (BF_10_ = 3.11, Supplementary Table S2).

**Table 3.** Number and percentage of participants that in each smell group that met the accuracy criteria for *SCENTinel 1.0*, along with group comparisons

Subtest	Anosmic individuals		Other smell disorders		Normosmic individuals		Total		BF_10_	Proportions		
	*N*	%	*N*	%	*N*	%	*N*	%		*X*^2^, df = 2	*P*	Significant comparisons
Odor detection	49	44	33	79	142	92	224	73	5.33e16 ± 0.01%	76.32	<0.001	a, b, c
Odor intensity	15	14	30	71	154	100	199	65	5.65e74 ± 0%	212.52	<0.001	a, b, c
Odor ID #1	36	34	32	76	142	92	212	69	3.55e24 ± 0.01%	102.61	<0.001	a, b, c
Odor ID #2	26	23	3	7	3	2	32	10	0.20 ± 0.03%	0.84	0.65	

Note: BF, Bayes factor for the model lmBF(subtest score ~ Group, data, which Random =‘ID’); ± X% (error of the estimate); df, degrees of freedom, *P*, *P* value, a, comparison anosmic individuals versus normosmic individuals; b, anosmic individuals versus other smell disorders; c, other smell disorders versus normosmic individuals; ID, identification.

**Figure 3.**
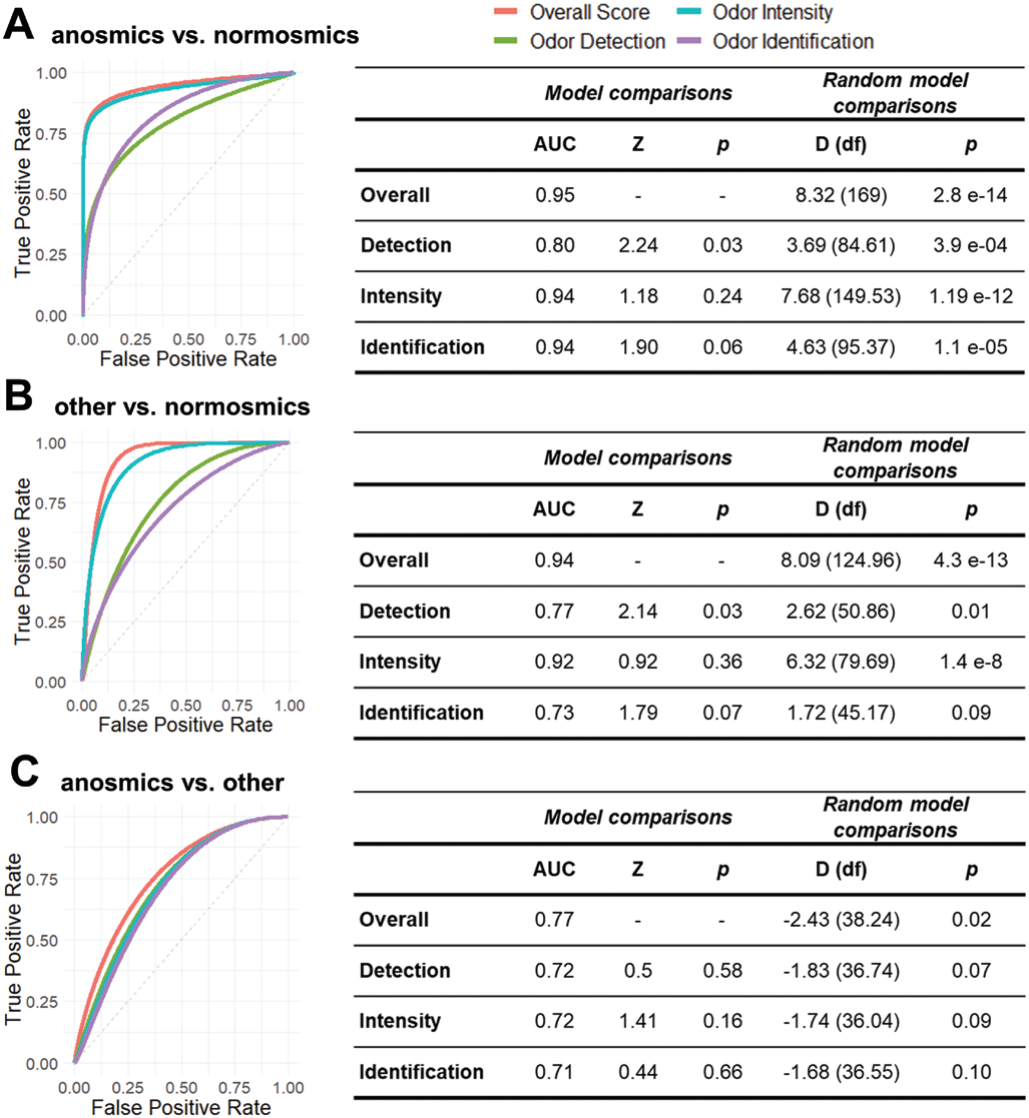
Receiver operating characteristic (ROC) curves and statistics on *SCENTinel 1.0* scores overall and for single subtests across groups: (**A**) anosmic individuals versus normosmic individuals; (**B**) other smell disorders versus normosmic individuals; (**C**) anosmic individuals versus other smell disorders) based on the linear discriminant analysis algorithm. AUC, area under the curve; *P*, *P* value; D, DeLong’s test for 2 ROC curves; df, degrees of freedom.

We then examined which classification algorithm would best predict belonging to a particular smell group. Results from a recursive feature selection indicated that 5 features (odor detection, intensity, identification, age, and female sex) recurred across samples. These results were confirmed by several other algorithms (Supplementary Figure S2). To assess whether *SCENTinel 1.0* subtests would be sufficient to discriminate between different groups, we investigated the ROCs that provided the greatest discrimination accuracy (LDA). As depicted in Figure 3, discrimination across the 3 smell groups is possible. The overall *SCENTinel 1.0* performance discriminated between anosmic and normosmic individuals with greater accuracy (AUC = 0.95) than any of the subtests alone (Figure 3A). The intensity subtest appears to be the single best discriminator between anosmic and normosmic individuals (AUC = 0.94), followed by odor identification #1 (AUC = 0.84) and odor detection (AUC = 0.80). Similarly, *SCENTinel 1.0* is also able to discriminate between individuals with other smell disorders and normosmic individuals (Figure 3B), as well as anosmic individuals versus individuals with other smell disorders (Figure 3C). In this latter comparison, AUC is greatly reduced (AUC = 0.77). As hypothesized, each *SCENTinel 1.0* subtest differently contributes to the classification of individual performance, and the contribution of each subtest to the classification is related to current smell ability. All *SCENTinel 1.0* subtests discriminate anosmic from normosmic individuals above chance, yet the overall *SCENTinel 1.0* performance does so with greater confidence (Figure 3A). Odor detection and intensity discriminate individuals with other smell disorders from normosmic individuals above chance, but odor identification does not (Figure 3B). Only the overall *SCENTinel 1.0* score discriminates above chance the performance of anosmic individuals from individuals with other smell disorders, whereas no subtest is able to do so in isolation (Figure 3C).

### Performance on *SCENTinel 1.0* and on the NIH Toolbox Odor Identification Test is comparable in normosmic individuals

**Figure 4.**
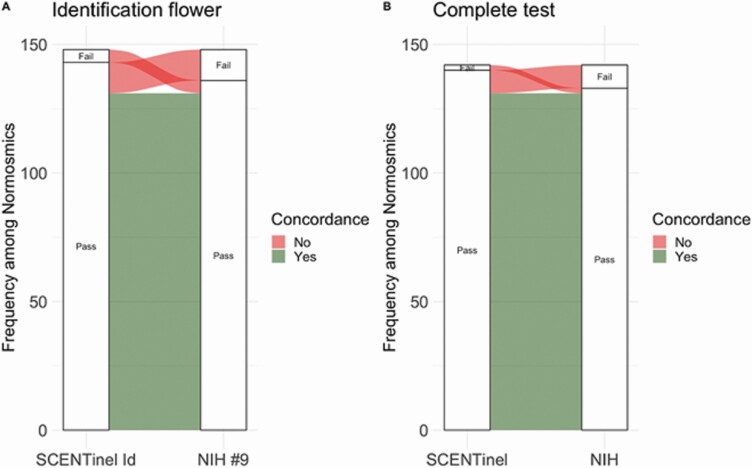
Concordance between *SCENTinel 1.0* and the NIH Toolbox Odor Identification Test in normosmic individuals: (**A**) concordance based on flower odor identification performance; (**B**) concordance based on full completion of both smell tests.

Normosmic individuals self-administered both *SCENTinel 1.0* and the NIH Toolbox Odor Identification Test to allow comparison of the performance of *SCENTinel 1.0* against a validated smell test. Results indicated that when comparing performance on the flower odor identification, which was odor #9 in the NIH Toolbox Odor Identification Test (143/148, 97% participants correctly identified the flower odor), and the *SCENTinel 1.0* odor identification subtest (136/148, 92% accuracy in the first identification attempt). A two-sample test for equality of proportions with continuity correction suggests the lack of statistical difference between the 2 test scores (*X*^2^ = 2.25, df = 1, *P* = 0.13). In 17/148 cases (12%), the NIH Toolbox Odor Identification Test and *SCENTinel 1.0* were discordant (Figure 4A, B, red ribbons); specifically, in 12 cases, the participant passed the NIH Toolbox Odor Identification Test but failed to meet the accuracy criteria for *SCENTinel 1.0*, and in 5 cases, the participant passed *SCENTinel 1.0* but failed the NIH Toolbox Odor Identification Test. When considering the full NIH Toolbox Odor Identification Test (9 items) and *SCENTinel 1.0* (detection, intensity, and identification, both attempts) the accuracy converged: 92% of normosmic individuals passed both tests. No effect of age (BF_10_ = 0.81 ± 0.02%), sex (BF_10_ = 0.84 ± 0.02%), or ethnicity (BF_10_ = 0.48 ± 0.02%) was found for the performance on the NIH Toolbox Odor Identification Test.

## Discussion

The goal of the present study was 2-fold: to assess the *SCENTinel 1.0* performance to discriminate conditions of ongoing smell loss and normosmia and to compare the performance of *SCENTinel 1.0* to the NIH Toolbox Odor Identification Test, a validated and standardized smell test. We hypothesized that normosmic individuals would meet the *SCENTinel 1.0* accuracy criteria at a higher rate than both the self-reported anosmic group and the group with other olfactory disorders and that normosmic individuals would perform similarly on *SCENTinel 1.0* and on the NIH Toolbox Odor Identification Test. Both of our main hypotheses were confirmed.

First, 94% of normosmic individuals met the *SCENTinel 1.0* accuracy criteria, in contrast to the 64% of participants reporting other smell disorders and only 10% of participants reporting anosmia. The majority of participants with anosmia were not able to meet the accuracy criteria for 2 or 3 subtests, particularly the odor intensity subtest. In comparison, participants with other smell disorders failed to meet the accuracy criteria for 2 subtests (especially the odor intensity subtest) more often than the normosmic group. Normosmic individuals met the accuracy criteria for all 3 subtests. The ability of the overall test to classify anosmic and normosmic individuals based on performance is satisfactory (AUC = 0.95). The odor intensity subtest alone has also a similar classification ability (AUC = 0.94), but ratings of intensity can be intentionally misreported, whereas the other subtests cannot. Odor identification represents the second-best subtest in discriminating between normosmic and anosmic individuals. Although this discrimination alone is less accurate compared with odor intensity, the odor identification subtest is an objective measure with a guessing probability of only 25% on the first attempt. Yet, the utility of the odor identification subtest is lost when discriminating normosmic individuals from those with other smell disorders. As anticipated, individuals suffering from hyposmia, which constitutes the majority of the other smell disorders group, may be able to report on odor quality but do not appropriately report its intensity. For individuals with parosmia, the performance could be different, yet these results cannot provide conclusive evidence given the low number of parosmic participants in this sample. Odor detection, which offers a culturally unbiased olfactory measure of olfactory performance (see, e.g., Doty et al. 2019), in concert with the other subtests aids the discrimination of anosmia from other forms of smell loss.

Second, we established that the normosmic individuals perform similarly for both the *SCENTinel 1.0* and the NIH Toolbox Odor Identification Test. Ninety-two percent of participants were able to meet the accuracy criteria of both full tests, and this figure increases to 97% when we consider the odor identification performance to a flower odor, which was the odorant tested here as well as the odor of item #9 in the NIH Toolbox Odor Identification Test.

We conclude that testing 3 olfactory functions with the goal of quickly detecting the presence of smell loss is possible and comparable to performance on the longer, validated and standardized NIH Toolbox Odor Identification Test, and is able to do so despite testing only one odorant at a time. *SCENTinel 1.0* meets all the scientific and practical criteria outlined above for population surveillance based on smell testing. Specifically, it is structured to reduce the probability of passing the test by guessing alone and to be self-administered. Due to the Lift’nSmell technology, no tools are needed to complete the test (e.g., a coin), and the intensity of the odorant is not affected by participant behavior (such as amount of surface scratched for scratch-and-sniff tasks). Altogether, this test can be applied in a variety of contexts and for different purposes.

The findings presented here represent the first step of a broader research program that includes a full validation and normative study on the *SCENTinel 1.0* test. Given our promising results and the urgent need to deploy all possible aids to control the spread of COVID-19, we report the data of this initial assessment. Presently, we have verified that *SCENTinel 1.0* is able to discriminate self-reported anosmic from normosmic individuals and that, among normosmic individuals, *SCENTinel 1.0* has been validated against the NIH Toolbox Odor Identification Test. Next, we are looking forward to extending testing to the multiple *SCENTinel* versions that feature multiple different odorants to assess whether performance is odor invariant (Zernecke et al. 2010). We are currently developing 8 versions of *SCENTinel 1.0*, which use non-trigeminal odors, highly familiar to the US population, as indicated by published data from existing databases (Freiherr et al. 2012; Dalton et al. 2013), and which achieve relative isointensity across a normosmic population. We also recognize the value of a test-retest evaluation for *SCENTinel 1.0*, comparison with a multiodorant test (NIH Toolbox Odor Identification test) among anosmic and normosmic populations, and collecting normative data across the life span, all of which are planned future efforts. Our goal is to achieve the highest fidelity in diagnosing smell alterations in the most rapid and least expensive method possible.

Then, we will focus our efforts on clinically verifying the diagnosis of smell disorders in patients, since at present participants self-report normosmia and/or the ongoing presence of smell disorders, including anosmia. If verifying the clinical diagnosis is a necessary step from a research perspective, olfactory routine testing with large-scale population deployment would likely lack this level of precision. It is therefore a very favorable result that *SCENTinel 1.0* can discriminate different degrees of self-reported olfactory ability in individuals without an in-depth research- or clinical-level investigation of their ability to smell. To this end, we intend to offer an analysis of the performance of *SCENTinel* across larger samples of individuals with hyposmia, parosmia, phantosmia, and so forth, to further our understanding of which olfactory functions have the most power in discriminating across smell disorders.

In the present study, we found no differences in performance based on age, sex, or ethnicity, relevant individual variables are known to affect olfactory performance (Hedner et al. 2010; Menon et al. 2013; Sorokowski et al. 2019). Although this initial study prominently featured women and White participants, unequally spread across different age groups, we aim at testing the performance of *SCENTinel 1.0* in more diverse groups to fully ascertain the effect of age, sex, and ethnicity and to identify possible cross-cultural and genetic influences that may play a role in test performance. Additionally, the brevity of *SCENTinel 1.0* can facilitate its translation and widespread use across linguistic communities (such as native Spanish and Chinese speakers). Further monitoring intraindividual performance over time will not only provide a path to better understanding recovery from smell loss but also offer the opportunity to determine a life span surveillance approach to olfactory perception, following in the footsteps of the NIH Toolbox Odor Identification Test, which can be used from 3 years of age with minimal modifications and from age 10 in its full form (Dalton et al. 2013).

Altogether, our findings support the idea that *SCENTinel 1.0* represents a rapid, accurate, flexible, and cost-effective tool to deploy a smell test in large-scale population surveillance efforts. The development of *SCENTinel 1.0* has been spurred by the new sudden loss of smell that characterizes COVID-19, including among nominally asymptomatic individuals, many of whom were not aware of their smell loss before receiving an objective olfactory test (Bhattacharjee et al. 2020; Gözen et al. 2021). The large-scale availability of a validated rapid smell test not only can benefit health emergencies such as COVID-19 but also can be used in early detection and monitoring of a variety of clinical conditions, including psychiatric, neurological, and neurodegenerative disorders.

## Supplementary Material

bjab012_suppl_Supplementary_Materials

## Appendix

### Eligibility survey

Thank you for taking the time to determine your eligibility for the validation of our smell test. Smell loss is one of the best predictors of COVID-19 and at present, rapid smell tests that can help monitor the spread of COVID-19 are not available. We have developed our 2-min smell test for this purpose.

We need your help to make sure that it is accurate and reliable.

We just have a few simple questions to determine your eligibility for this study.

Please specify your sex.

❏ Male
❏ Female
❏ Other: _____________
❏ Prefer not to answer

Do you currently reside in the United States?

❏ Yes
❏ No

I am between 18 and 75 years old.

❏ Yes
❏ No

Do you currently have a confirmed or diagnosed smell or taste disorder?

❏ Yes
❏ No

If yes, please specify:

❏ Anosmia (complete loss of smell)
❏ Hyposmia (partial loss of smell)
❏ Parosmia (the quality of some odors has changed)
❏ Fluctuations (sometimes I can smell, sometimes I
❏ cannot)
❏ Other: _______________________

I have access to a smart device (e.g., cell phone, computer, etc.).

❏ Yes
❏ No

-------------------------------------------------------------------------------------------------------------------------------

You are not eligible to participate in the study.

We immensely appreciate your willingness to help us validate our Rapid Smell Test used for COVID-19 surveillance.

Thank you for your time.

-------------------------------------------------------------------------------------------------------------------------------

Thank you for your time.

You are eligible to participate in the study!

We immensely appreciate your willingness to help us validate our Rapid Smell Test used for COVID-19 surveillance.

For this study, you will receive in the coming days/weeks an envelope with the testing materials.

You will be asked to complete our Rapid Smell Test, and/or another validated smell test, the NIH Toolbox, as well as answer some questions about you and your sense of smell online.

Overall your participation will take max ~10–15 min. We ask you to kindly complete the test by 3 September 2020.

Do you wish to enroll in the study?

❏ Yes
❏ No

Please provide your mailing address to receive the smell testing materials (First and Last Name, Street, City, State, ZIP). __________________________________________

I would like for Monell to keep my mailing address so that I receive the Center’s correspondence.

❏ Yes
❏ No

Are you confident that you would be able to complete the smell test prior to 3 September 2020? It will only take ~10–15 min maximum.

❏ Yes
❏ No

The following question is completely optional and will not be used to determine eligibility. Since the intended use of this smell test is to provide a surveillance tool for COVID-19, it is important that we compare it against COVID-19 diagnostic test results (both positive and negative).

(Optional) Would you be willing/have access to receive a COVID-19 nasal-swab test in the next couple of weeks (with or without the presence of symptoms)?

❏ Yes
❏ No
❏ Unsure

Thank you for taking the time to complete our survey.

You will receive in the coming weeks mail from Monell with the testing material.

Stay tuned!
