# Supplementary material for: SCENTinel 1.0: Development of a Rapid Test to Screen for Smell Loss
Source: Chem Senses. 2021 Mar 27;46:bjab012. doi: 10.1093/chemse/bjab012 (PMC8083606; doi:10.1093/chemse/bjab012)
Supplement: bjab012_suppl_Supplementary_Materials [file bjab012_suppl_supplementary_materials.docx]

**Supplementary Materials**

**Figure S1.** Prototype of the letter sent to participants that includes *SCENTinel 1.0*. Shown is a draft customizable card at the moment. Further design development is under way.

**
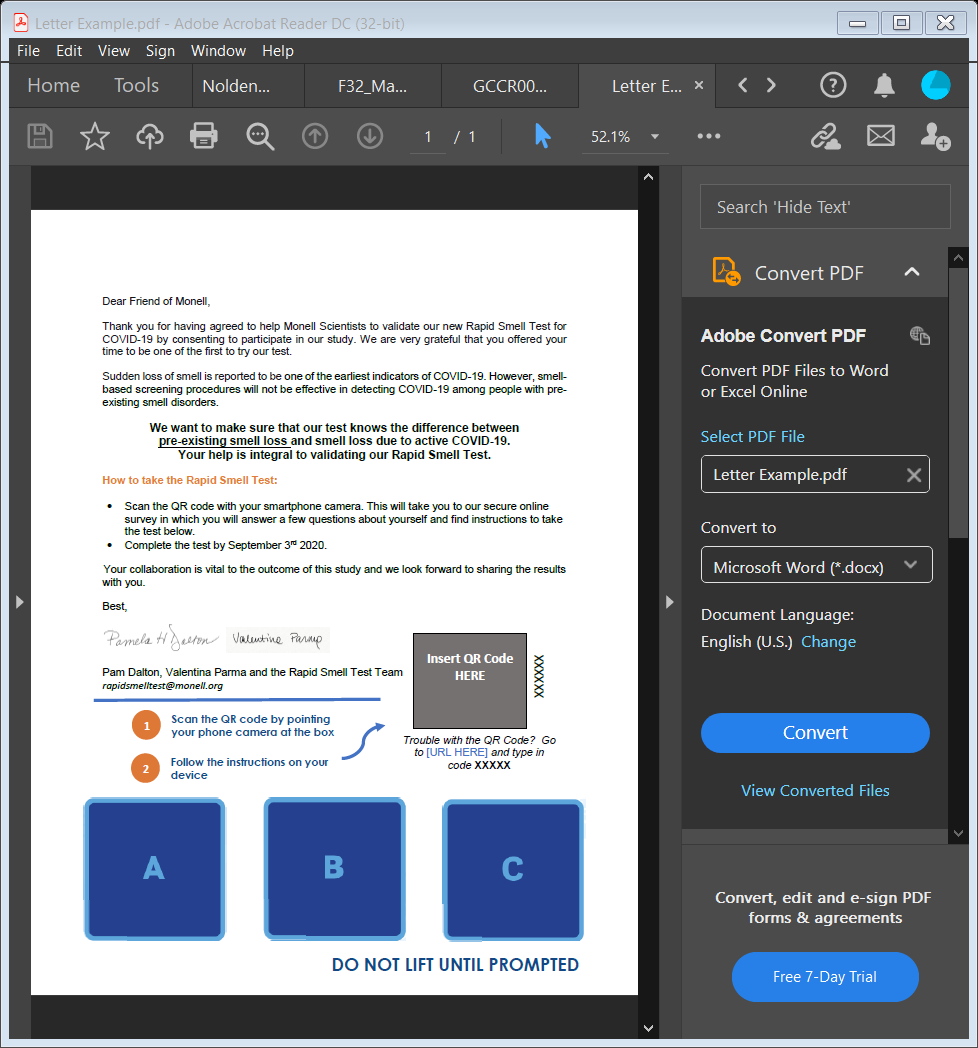
**

**Table S1.** Number and percentage of participants who used a specific response pattern to complete *SCENTinel* 1.0

| Response Pattern # | Anosmics | | Other Smell Disorders | | Normosmics | | Total | |
| --- | --- | --- | --- | --- | --- | --- | --- | --- |
|  | N | *%* | N | *%* | N | *%* | N | *%* |
| 1 | 7 | *6* | 22 | *52* | 139 | *96* | 168 | *44* |
| 2 | 12 | *11* | 7 | *17* | 0 | *0* | 19 | *6* |
| 3 | 0 | *0* | 1 | *2* | 2 | *1* | 3 | *1* |
| 4 | 10 | *9* | 0 | *0* | 0 | *0* | 10 | *3* |
| 5 | 0 | *0* | 3 | *7* | 1 | *1* | 4 | *1* |
| 6 | 20 | *19* | 0 | *0* | 0 | *0* | 20 | *7* |
| 7 | 4 | *4* | 1 | *2* | 3 | *2* | 8 | *3* |
| 8 | 15 | *14* | 2 | *5* | 0 | *0* | 17 | *6* |
| 9 | 2 | *2* | 0 | *0* | 0 | *0* | 2 | *1* |
| 10 | 14 | *13* | 2 | *5* | 0 | *0* | 2 | *1* |
| 11 | 2 | *2* | 3 | *7* | 9 | *6* | 14 | *5* |
| 12 | 25 | *23* | 1 | *2* | 0 | *0* | 26 | *8* |

Grey shaded row: accurate response patterns; # = response pattern number.

**Table S2.** Assessment of the effect of demographic covariates on the *SCENTinel 1.0* subtests.

| **Subtests** | **Demographic variable** | **BF10** |
| --- | --- | --- |
| **Odor Detection** | **Age** | 0.18 ± 0.01% |
|  | **Sex** | 0.31 ± 1.37% |
|  | **Ethnicity** | 0.14 ± 0.97% |
| **Odor Intensity** | **Age** | 0.33 ± 0.01% |
|  | **Sex** | 0.18 ± 1.22% |
|  | **Ethnicity** | 0.15 ± 1.05% |
| **Odor Identification #1** | **Age** | 3.11 ± 0.01% |
|  | **Sex** | 0.27 ± 2.32% |
|  | **Ethnicity** | 0.13 ± 2.22% |
| **Odor Identification #2** | **Age** | 0.11 ± 0.01% |
|  | **Sex** | 0.25 ± 1.19% |
|  | **Ethnicity** | 0.92 ± 7.37% |

BFAge = main effect model; BFSex and BFEthnicity = BFSex(Ethnicity)+Group/BF Group. The additive models represented the best models in all comparisons tested.

**Figure S2. A.** Prediction metrics for five algorithms used to predict multiclass group belonging (anosmics, other smell disorders, normosmics) based on the performance at *SCENTinel 1.0*. **B.** Feature importance based on the LDA model across the three smell groups.

**A.**

| **Algorithm** | **AUC** | **Sensitivity** | **Specificity** | **PPV** | **NPV** |
| --- | --- | --- | --- | --- | --- |
| **LDA** | 0.95 | 0.73 | 0.95 | 0.89 | 0.86 |
| **Elastic Net** | 0.95 | 0.77 | 0.92 | 0.85 | 0.88 |
| **SVM Linear** | 0.94 | 0.82 | 0.87 | 0.78 | 0.89 |
| **Random Forest** | 0.93 | 0.73 | 0.92 | 0.84 | 0.85 |
| **SVM Radial** | 0.92 | 0.59 | 0.92 | 0.81 | 0.80 |

Note: LDA: Linear Discriminant Analysis; SVM: Small Vector Machine.





**B.**
